# Novel image markers for non-small cell lung cancer classification and survival prediction

**DOI:** 10.1186/1471-2105-15-310

**Published:** 2014-09-19

**Authors:** Hongyuan Wang, Fuyong Xing, Hai Su, Arnold Stromberg, Lin Yang

**Affiliations:** Department of Statistics, University of Kentucky, 725 Rose Street, 40536 Lexington, KY USA; J. Crayton Pruitt Family Department of Biomedical Engineering, University of Florida, 1275 Center Drive, 32611 Gainesville, FL USA; Department of Electrical and Computer Engineering, University of Florida, 233 Larsen Hall, 32611 Gainesville, FL USA; Department of Computer Science, University of Kentucky, 329 Rose Street, 40536 Lexington, KY USA

**Keywords:** Lung cancer, Segmentation, Classification, Image informatics, Survival analysis

## Abstract

**Background:**

Non-small cell lung cancer (NSCLC), the most common type of lung cancer, is one of serious diseases causing death for both men and women. Computer-aided diagnosis and survival prediction of NSCLC, is of great importance in providing assistance to diagnosis and personalize therapy planning for lung cancer patients.

**Results:**

In this paper we have proposed an integrated framework for NSCLC computer-aided diagnosis and survival analysis using novel image markers. The entire biomedical imaging informatics framework consists of cell detection, segmentation, classification, discovery of image markers, and survival analysis. A robust seed detection-guided cell segmentation algorithm is proposed to accurately segment each individual cell in digital images. Based on cell segmentation results, a set of extensive cellular morphological features are extracted using efficient feature descriptors. Next, eight different classification techniques that can handle high-dimensional data have been evaluated and then compared for computer-aided diagnosis. The results show that the random forest and adaboost offer the best classification performance for NSCLC. Finally, a Cox proportional hazards model is fitted by component-wise likelihood based boosting. Significant image markers have been discovered using the bootstrap analysis and the survival prediction performance of the model is also evaluated.

**Conclusions:**

The proposed model have been applied to a lung cancer dataset that contains 122 cases with complete clinical information. The classification performance exhibits high correlations between the discovered image markers and the subtypes of NSCLC. The survival analysis demonstrates strong prediction power of the statistical model built from the discovered image markers.

## Background

Lung cancer is one of the most frequent cancers worldwide. Similar to breast cancer in female, lung cancer is the leading cancer in males, with 17% of the total new cancer cases and 23% of the total cancer deaths. The prognosis of lung cancer is still poor, with five-year survival rate of approximately 10% in most countries. Lung cancer can be classified as small cell lung cancer (SCLC) and non-small cell lung cancer (NSCLC). NSCLC accounts for the majority (84%) of lung cancer [1]. Two major types of NSCLC are adenocarcinoma (including bronchi alveolar carcinoma) representing about 40% and squamous cell carcinoma representing about 25–30% [2]. Accurate classification and survival analysis can provide assistance for personalized treatment planning and prognosis.

**Figure 1:**
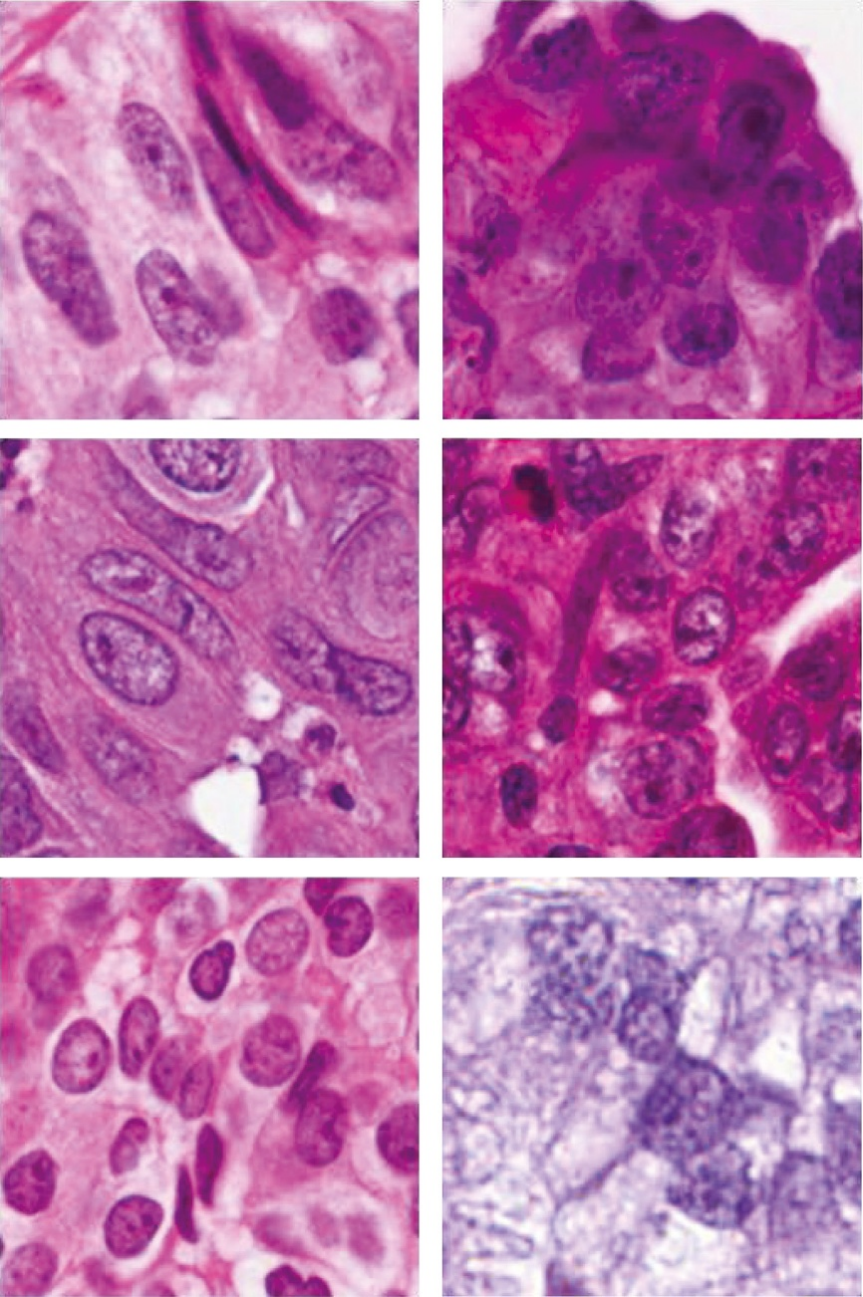
**Example Images of squamous cell carcinoma (Left) and adenocarcinoma (Right).** Notice: 1. Elongated or spindle cells are more abundant in squamous; 2. Squamous has more clear cell boarders; 3. Squamous usually are more pink while Adeno are more purple or blue.

Histopathology images serve as a golden standard for lung cancer diagnosis since they can provide a comprehensive view of the disease and its effect on human tissue [3]. Figure 1 shows some representative images of squamous cell carcinoma and adenocarcinoma. Currently, pathologists make diagnosis decision based on cellular and inter-cellular level morphology. Most of current pathology diagnosis is still based on subjective opinions of pathologists and the varying abilities of doctors could result in large interpretation errors or bias. The proposed framework, which focuses on automated quantitative analysis of histopathology images, could alleviate the subjectivity in NSCLC diagnosis and provides supports to doctors in lung cancer classification and patients’ survival analysis.

Recently, there are much active research in imaging informatics [4–12]. Before computer-aided lung cancer diagnosis and survival analysis, usually accurate image segmentation [13, 14] is a prerequisite. Sometimes the explicit segmentation may not be required for the applications when the tumor microenvironment is critical for tumor classification; however, in our study, we find that explicit cell localization and cellular features are important for NSCLC classification and survival analysis.

Because crowding and overlapping cancer cells often present significant challenges for most traditional automatic segmentation methods. A vast variety of algorithms based on watershed and its variants [15–17], graph cut [18, 19], and active contour models [20–22] have been proposed. However, none of these methods could robustly handle touching cell segmentation challenges exhibited in lung cancer images. Lu et al. [23] has proposed a supervised learning-based segmentation algorithm to support new image features extraction and polyp detection on CT images, and a flexible, hierarchical feature learning framework integrating different levels of discriminative and descriptive information is presented in [24]. Supervised learning is a potential approach to tackle these challenges, but it requires a lot of labeled training data provided by experienced pathologists. For computer-aided classification, genetic algorithms (GAs) and support vector machines (SVMs) have been combined for multi-class cancer identification based on microarray dataset [25]. Partial least square regression (PLSR) and support vector machine with recursive feature elimination (SVM-RFE) have been applied to lung cancer subtype classification [26]. In [27], the lung cancer image classification is modeled as a multi-class multi-instance learning problem, and an adaboost algorithm has been used to perform classification with a bag of feature model. None of these studies correlated image features with the patient survival information.

Survival analysis is related to death in biological organisms and failure in mechanical systems. Several commonly used survival analysis methods are the Kaplan-Meier method for estimating the survival function [28], the log-rank test for comparing the equality of two or more survival distributions, and the Cox proportional hazards (PH) model for examining the covariate effects on the hazard function [29]. In survival analysis, one important issue that needs to be considered is censoring problem (subjects are censored if they are not followed up or if the study ends before they die or have an outcome of interest). Cox proportional hazards model [30] is one of the most commonly used multivariate approaches to analyze the survival time data in medical research. It is a semi-parametric method that does not need a specific baseline hazard function and has the capability to effectively handle censoring problem. In other words, it is not necessary to specify a survival distribution to model the effect of the explanatory variables on the duration variable.

In survival analysis, researchers also considers clinical factors or other environmental information [29, 31–33]. For example, standard Cox proportional hazards survival model with a spatial random effect extension has been applied and proved that the long-term exposure to combustion-related fine particulate air pollution is an important environmental risk factor for cardiopulmonary and lung cancer mortality [31]. Gene signature expressions have also been used as covariates to conduct survival analysis [34–39] to search for pairs of genes (biomarkers) that are significantly related to patient death.

In this paper, we will present an integrated framework that investigates the prognostic effects of image markers. First, a novel seed detection-based repulsive deformable model is proposed to separate touching cells; secondly, a set of geometry, pixel intensity, and image texture features are extracted to describe cellular morphological properties; thirdly, eight different classification techniques are comparatively analyzed for computer-aided diagnoses of NSCLC; finally, survival analysis is conducted based on a Cox model fitted by component-wise likelihood based boosting. The entire system is designed to assist doctors for more objective and accurate diagnoses and prognoses of NSCLC. Unlike gene sequencing, histopathological slides are always available for each lung cancer patient in routing clinical diagnosis, and therefore the adoption of the developed prediction model does not require any changes to current clinical practice.

The experiments in the paper are conducted using the adenocarcinoma and squamous cell carcinoma lung cancer images downloaded from the TCGA Data Portal. TCGA (The Cancer Genome Atlas) is a collection of cancer specimens, with additional clinical information about participants, metadata about the samples, histopathology slide images from sample portions and molecular information derived from the samples. It is supervised by National Cancer Institute (NCI) and National Human Genome Research Institute (NHGRI) and freely available to researchers.

## Methods

### Cell detection and segmentation

Seed detection is the first critical step for marker-based segmentation methods. Motivated by [22], we have proposed a multi-scale distance map-based voting algorithm for cell detection. The newly developed method can efficiently handle relatively large cell size and shape variation. For each point (*x*,*y*), we define a cone-shape voting area *A* with vertex at (*x*,*y*) and votes towards the negative gradient direction of the vertex. A 2D Gaussian kernel *K*(*m*,*n*,*μ*,*Σ*) is introduced to weight the voting points:1

where *C*_1_ is the normalized constant, *S* represents the set of all voting points, *A*_*c*_(*m*,*n*) denotes the cone-shape voting area with vertex (*m*,*n*) and scale *c*. The voting area at each scale is defined as the radial range (*r*_*min*_,*r*_*max*_) and angular range *Δ*,  (*θ* is the angle of the gradient direction with respect to *x* axis) is the mean of the Gaussian kernel. *Σ*=*σ*^2^*I*_2_ (*I*_2_ is the identity matrix) is the covariance matrix.  is the indicator function: 1 for (*x*,*y*)∈*A*_*c*_(*m*,*n*) and 0 otherwise. *gD*(*x*,*y*,*σ*) represents the Euclidean distance map. After the confidence map *V*(*x*,*y*) is calculated, we remove those points with relatively lower voting values, which locate near the cell boundaries. In order to achieve a robust seed detection, we apply mean shift [40] to locate the final positions of cell seeds.

Using the boundaries of detected cell seeds as initializations, a novel repulsive balloon snake (RBS) algorithm based on a deformable model [41] is used to seek the cell boundaries. RBS is a parametric model which can naturally preserve cell topologies and prevent contours from splitting or merging with one another.

A snake is an active curve as *v*(*s*)=(*x*(*s*),*y*(*s*)),*s*∈[0,1], moving through the image domain to minimize its energy functional, under the influence of internal and external forces. To enforce snakes to inflate or deflate, a pressure force to propose the balloon snake (BS) model was introduced [41]. The external force  is calculated by:2

where ***n***(*s*) represents the normal vector (pressure force) to the curve at the specific point on *v*(*s*) and ∇*E*_*ext*_(*v*(*s*)) is defined as image force, where *E*_*ext*_(*v*(*s*))=-||∇*I*(*v*(*s*))||^2^ (*I*(*v*(*s*)) is the original image). *γ* and *λ* are the weighting parameters controlling pressure force and image force, respectively.

Balloon snake (BS) model can not be directly used for touching object segmentation. If all balloon snakes move independently, they will cross with one another. Based on these observations, we introduce an interactive scheme to form a RBS model for touching cell segmentation. The intrinsic idea of RBS is based on the following: the cell contour should be driven by its own forces as well as extrinsic forces from other deformable contours; both amplitudes and directions of these extrinsic forces should vary with respect to the distance between snakes. When two snakes are far away, their movements should be dominantly controlled by their own driven forces (internal and external forces); when they get closer, each snake should receive repulsive forces from all other adjacent snakes. As a result, the extrinsic force will prohibit snakes from crossing or merging.

Given an image *I* with *N* cells (denoted by *N* curves *v*_*i*_,*i*=1,…,*N*), the new repulsive external force  for *v*_*i*_ is defined as:3

where *d*_*ij*_(*s*,*t*)=||*v*_*i*_(*s*)-*v*_*j*_(*t*)||_2_ is the Euclidean distance between *v*_*i*_(*s*) and *v*_*j*_(*t*). *f*(*x*)>0,*x*∈(0,+*∞*), represents a monotonic decreasing function (*f*(*x*)=*x*^-2^ in our case), and *ω* weights the repulsive force. For a specific point *v*_*i*_(*s*), the closer it moves to other snakes, the more repulsive forces it will receive. Unlike the original balloon snake, RBS moves contours under the influence of their own driven forces and extrinsic repulsive forces from other snakes. When these two types of forces achieve a balance, snakes stop evolving.

### Feature extraction

Based on the segmented cell boundaries, three groups of cellular features are extracted for subsequent classification and survival analysis. In total we have extracted 166 image morphometric features, which are represented as the candidates of image markers. The detailed notations of feature names and descriptions are listed in the Table 1.

**Table 1:** **The image features and their descriptions**

Name	Description
area1-6	Cell area feature
axis1-6	Major-minor axis ratio
cir1-6	Cell circularity feature
peri1-6	Contour perimeter feature
solidity1-6	Contour solidity feature
mean1-6	Cell intensity mean feature
std1-6	Cell intensity stand deviation feature
kurt1-6	Cell intensity Kurtosis
entr1-6	Cell intensity entropy
energy1-6	Cell intensity energy
contrast1-6	Cell intensity contrast
corr1-6	Cell intensity correlation
engy1-6	Cell intensity energy from co-occurrence matrix
homo1-6	Cell intensity homogeneity
skew1-6	Cell intensity skewness
tfcm1-24	Texture feature coding method (TFCM)
csac1-24	Center symmetric auto correlation (CSAC) feature
lbp1-24	Local binary pattern (LBP) feature
t1-4	Texton histogram feature

The 1-6 in each image feature represent the mean, median, variance and three frequency values of the histogram for each intensity and geometric feature, respectively. Csac, tfcm, lbp and texton histogram features are high dimensional feature vectors, therefore we calculate their moment statistics to reduce the dimensionality. In total we have extracted 166 geometric, pixel intensity, and image texture feature variables for each patient. All variables are normalized before further classification and survival analysis.

**Group 1: Geometry Features.** Five geometry features are calculated for each segmented lung cancer cell, including area *A*_*cell*_, contour perimeter *P*_*cell*_, circularity , major-minor axis ratio, and contour solidity that is defined as the ratio of cell area region over the convex hull defined by the cell boundary.

**Group 2: Pixel Intensity Statistics.** This group of features are calculated based on the pixels in the segmented cells, including intensity mean, standard deviation, skewness, kurtosis, entropy, and energy. We use *Lab* color space for better color representation.

**Group 3: Texture Features:** This group of features contains co-occurrence matrix [42], local binary pattern (LBP) [43], texture feature coding method (TFCM) [44], center symmetric auto-correlation (CSAC) [45], and texton features [46]. The co-occurrence matrix [42] is an estimation of the joint probability distribution of intensity of two neighboring pixels. LBP [43] is a measure of local textures. Each pixel in the input image patch is assigned a binary code by comparing the intensity of this pixel to those of its neighbors. Similar to LBP, in TFCM [44], each pixel is assigned a texture feature number (TFN). The TFN of one pixel is generated by comparing this pixel with its neighbors in four directions: 0°, 45°, 90°, and 135°. A histogram is calculated based on the TFNs of one image patch. CSAC is a measure of the local patterns with symmetrical forms. We calculated a series of local auto-correlation and covariance introduced in [45], including symmetric texture covariance (SCOV) and variance (SVR), and within-pair variance (WVAR) and between-pari variance (BVAR). 3×3 pixel unit of each channel is considered for CSAC feature. Texton [46] is a discriminative texture representation. The calculation of texton feature is based on unsupervised learning. We randomly picked some cells in each image as training samples. These cell patches are filtered by texton filter bank. K-means clustering is then applied and the centers of the clusters are defined as textons. To generate the texton histogram for a new image, the image is first filtered by the same texton filter bank, then each pixel is assigned to one texton to build the final texton histogram.

### NSCLC classification

After calculating the aforementioned image features, we first perform the NSCLC subtype classification. In this stage, several conventional machine learning methods and recently published state-of-the-art algorithms that can handle high dimensional data are compared, which include multiple support vector machine recursive feature extraction (MSVM-RFE) algorithm [47], L1 penalized logistic regression [48, 49], random forest [50, 51], naive Bayesian [52, 53], adaboost [54, 55], sparse coding spatial pyramid matching (ScSPM) alogrithm [56], locality-constrained linear coding (LLC) [57], and nearest class mean (NCM) classifier [58]. MSVM-RFE is an iterative feature selection method that uses a backward elimination procedure. Resampling scheme is introduced to stabilize the feature rankings. At each iteration, the feature ranking score is computed based on the weight vectors of multiple linear SVMs trained on subsamples of the original training data and the feature with the smallest ranking score is removed from the model. L1 penalized logistic regression provides an efficient lasso regularization path for logistic regression, which enables feature shrinkage and selection for high dimensional data. Random forest is an ensemble learning method for classification, which can generate a score by permutation to rank the importance of variables in classification problem. Naive Bayesian classifier is a simple probabilistic classifier based on the Bayes theorem with naive feature conditional independence assumptions. The adaboost algorithm employs the idea of sequentially applying a classification algorithm to reweighted versions of the training data and then taking a weighted majority vote of a ensemble of weak classifiers. Adaboost can provide an importance score for each weak classifier that corresponds to one selected feature. ScSPM is an extension of spatial pyramid matching [59] and the algorithm uses selective sparse coding followed by multi-scale spatial max pooling and SVM. LLC is another feature representation method that applies locality constraint to project each feature into a sparse code. NCM is a distance-based classification with projecting the features into a low-dimensional space for classification. These three recent algorithms have already made remarkable successes on a range of nature image classification benchmarks.

### Survival analysis

Before survival analysis, dimension reduction is a widely used approach to avoid the “curse of dimensionality”. Common examples of linear dimension reduction methods, such as principal component analysis (PCA), are proposed to minimize the variances. Meanwhile, least absolute shrinkage and selection operator (LASSO) [60] method is another classic method of feature shrinkage and selection for regression that can potentially handle high dimensional data. Least angle regression (LARS) is proposed for variable selection in the linear regression setting for high dimensional data [61]. The LARS selects predictors by its current correlation or angle with the response, where the correlation is defined as the co-correlation between the predictor and the current residuals. Moreover, elastic net is proposed as a new regularization and variable selection method for feature selection [62]. Boosting is another widely used feature selection approach. It applies the idea of fitting an ensemble of weak learners to the data. Furthermore, component-wise boosting has been proposed to estimate the model with intrinsic variable selection [63]. The term component-wise means each base learner only consists of linear function of one component (variable). For each covariate, a base-learner is specified and only the best base-learner is updated in each boosting step. Finally only part of base learners are chosen to ensemble the strong classifier when the optimal boosting iteration is reached. The algorithm can generate a strong classifier and a sparse set of selected features.

Given the observations (*t*_*i*_,*d*_*i*_,*x*_*i*_),*i*=1,2,…,*n*, where *t*_*i*_ is the observed time to the event of interest for individual *i*, *d*_*i*_ equals 1 if an event occurred at that time and 0 if the observation has been censored, and *x*_*i*_ is vector of covariates obtained at time 0. The component-wise likelihood based boosting algorithm for high dimensional survival analysis is based on the Cox proportional hazards model:4

where *λ*_0_(*t*) is the baseline hazard and *x* is covariate vector. For estimation, the baseline hazard *λ*_0_(*t*) is left unspecified and the estimate of *β* is obtained by maximizing the partial log likelihood.5

For high dimensional data, penalized regression methods like LASSO [60], ridge regression [64], would add a penalization term into the partial log likelihood function and the penalized partial likelihood is maximized by techniques such as quadratic programming.

In this paper, we apply component-wise likelihood based boosting [65] to dimensionality reduction, which adapts from the offset-based boosting approach from [66]. In each iteration the previous boosting steps are incorporated as an offset in a penalized partial likelihood estimation. Component-wise indicates that only one single parameter, i.e., one covariate, is updated in every iteration by maximization the L2 penalized partial log likelihood with respect to each candidate covariate.6

where *I*_*p*_ is a diagonal matrix to penalize each covariate separately, with diagonal elements equal to 1 for each candidate and 0 for the rest corresponding to penalization and no-penalization. The candidate covariate that can best improve the overall fitting will be selected for updating. As the number of boosting steps increases, more feature variables will be selected and chosen with respect to their relevances in predicting survival rates. The result is expected to be sparse with many coefficients equal to zero. The coefficient paths of component-wise boosting are expected to be more stable than LASSO based approaches [65]. In addition, it has two major advantages over LASSO: 1) it allows for unpenalized mandatory covariates; 2) it can handle correlated covariates by including pathway information [67].

## Results

### Cell detection and segmentation

**Figure 2:**
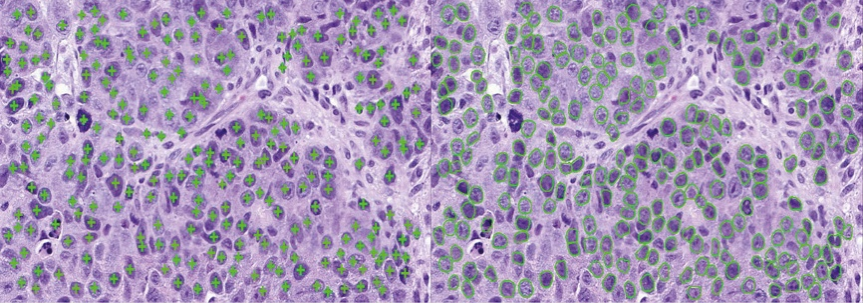
**Cell detection (left) and segmentation (right) results.** Please note that the cells with small areas correspond to non-tumor cells, and are automatically removed after the boundaries are extracted. Those false detected seeds locating in the lymphocyte regions are also removed using a simple intensity threshold before cell segmentation is conducted.

The cell detection and segmentation results are displayed in Figure 2. It can be observed that even for heavily touched regions, cells are still accurately detected and segmented automatically. It is worth mentioning that the proposed cell detection algorithm can handle relatively large size variations, and the repulsive snake models can prevent contours from overlapping with one another.

We have compared the proposed voting method (SPV) presented in [22] and the phase-coded hough transform (HT) based on quantitative measurement. In our evaluation a positive detection is counted if a detected seed locates within a 8-pixel circle around a ground truth seed; otherwise, a miss is counted. To measure the accuracy of the cell detection algorithms, we compute the mean, variance, maximum and minimum of the distance between the detected seeds and their corresponding ground truth seeds. In addition, we also show miss rate (MR) and false positive rate (FP) in Table 2. The ground truth seeds are manually generated for comparison. As one can see, the improved voting approach produces the best performance in comparison with other two methods.

**Table 2:** **The pixel-wise seed detection accuracy compared with ground truth**

	Mean	Variance	Max	Min	MR	FP
HT	3.9	4.13	8.0	0.19	0.46	0
SPV	3.0	3.13	7.9	0.29	0.21	0.002
Proposed	**2.6**	**2.8**	7.9	**0.12**	**0.12**	0.002

The best performance measured in each metric is marked in boldface.

To evaluate the performance of the segmentation algorithm, we define precision  and recall , where *seg* represents the segmentation result and *gt* represents the manually-generated ground truth. We show the mean, variance and 80% in Table 3. The segmentation algorithm can effectively handle touching cells and provide accurate segmentation results with high precision and recall rates.

**Table 3:** **The performance of segmentation measured by precision and recall**

Precision	Precision	80%	Recall	Recall	80%
mean	variance		mean	variance	
0.87	0.01	0.95	0.95	0.01	0.96

### NSCLC classification

Precison, recall, and accuracy have been used as prediction performance metrics for NSCLC classification. The training (62) and testing (60) datasets have been randomly selected and repeated five times to test the accuracies of classification using the TCGA dataset consisting of 122 NSCLC patients. Table 4 shows the average recall, precision, and accuracy using eight classifiers. The experiments indicate that the random forest and adaboost provide the best results.

**Figure 3:**
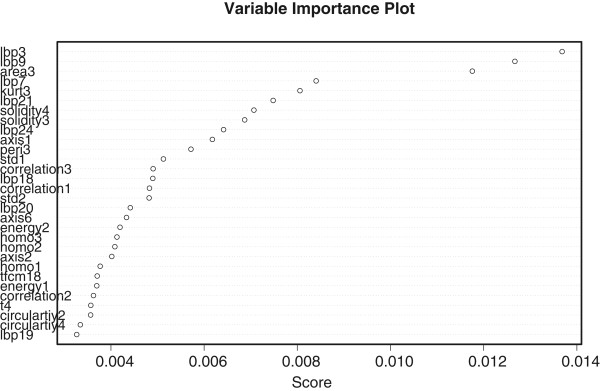
**The feature importance of the 166 image morphometric features.** Only the top 30 image features are shown here. The *x*-axis represents the frequency score and *y*-axis denotes the name of each feature variable.

To assess the relative importance of the 166 image markers, we have applied adaboost to the entire dataset and generated the frequency score for the variable selected in each boosting iteration. The results are shown in Figure 3. Higher importance score indicates a more representative feature variable for NSCLC classification.

**Table 4:** **The average recall, precision and accuracy of NSCLC classification**

	MR	PL	NB	RF	AB	LLC	SC	NCM
Recall (*%*)	89.7	75.9	71.0	93.1	92.4	75.3	66.1	74.1
Precision (*%*)	80.5	55.9	67.3	90.6	90.9	80.4	83.2	75.4
Accuracy (*%*)	84.3	53.6	66.4	92.0	91.7	76.7	69.7	72.3

MR: MSVM-RFE, PL: L1 penalized logistic regression, NB: naive Bayesian, RF: random forest, AB: adaboost, LLC: locality-constrained linear coding, SC: sparse coding spatial pyramid matching, NCM: nearest class mean classifier.

The top 10 features selected by adaboost are 4 lbp features, 3 solidity features, and area3, kurt3 and peri3 features. Among the top 30 features, lbp, area, solidity, axis, tfcm, energy, correlation and contrast are most commonly selected image features. Peri, kurt, std and circularity all have one feature been selected. The ranking suggests that the image texture features and geometric features are representative markers to distinguish between two subtypes of NSCLC: Adenocarcinoma (AC) and Squamous cell carcinoma (SCC). The results also indicate that 1) there are more elongated cells for SCC than AC; 2) AC usually has a relatively larger intensity variation inside cells than SCC; 3) SCC cells are often over-stained and exhibit more clear boundaries; 4) AC cells usually exhibit more inhomogeneous texture than SCC.

### Survival analysis

**Figure 4:**
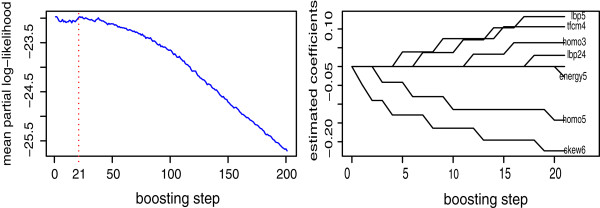
**(Left) The plot of mean partial log likelihood against the number of boosting steps using six-fold cross validation and (Right) the plot of coefficient paths, i.** **e. the parameter estimation plotted against the boosting steps until the optimal steps were reached.** M = 21 is used since it minimizes the mean partial log likelihood. Since we use an offset-based update mechanism, the same image feature might be reselected and its coefficient would be updated. We use branch-out for illustration purpose when one specific image feature is selected.

The TCGA NSCLC dataset contains complete patients’ histopathological image information. It has been randomly divided into training (*n*=65) and testing set (*n*=57). The training set is used to build a Cox regression model with component-wise likelihood based boosting for feature selection. Among 166 image features, we first conduct univariate Cox regression and abandon those with Wald test *p* value less than a threshold (0.25). The rest image features are chosen as candidate markers to conduct component-wise likelihood based boosting for Cox Proportional Hazards Model. After the univariate Cox regression step, 59 image features have been selected as candidates. The penalty parameter *λ*, which determines the size of the boosting steps, is determined based on cross validation. Six-fold cross validation on the training set has been performed to choose the number of boosting steps *M* (Figure 4). The final representative image markers selected are energy5, lbp5, lbp24, homo3, homo5, tfcm4 and skew6 with corresponding coefficients -0.0268, 0.1670, 0.0343, 0.0685, -0.1382, 0.1130 and -0.2150. Please note that all these feature covariates selected belong to pixel intensity features and texture features. This demonstrates that cell staining and inhomogeneity inside the nuclei, which may indicate the protein structures of the cancer cells, hold strong potential to predict NSCLC patients’ survival.

After the prediction model training procedure, we have employed the time dependent ROC curves for uncensored data and AUC as criteria to select the best thresholds for risk scores and assess how well the model predicts patients’ survival outcome. At time *t*, larger AUC indicates better predictability of time to event measured by sensitivity and specificity. After classifying patients into low- and high-risk groups, we can estimate and compare their Kaplan-Meier survival curves. The performance of such a binary classifier is generally evaluated in terms of the overall predictive accuracy.

**Figure 5:**
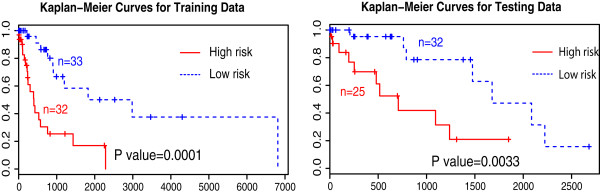
**Kaplan-Meier survival curves of two groups classified by predicted risk scores for training (Left) and testing dataset (Right).** The *p* value of log rank test for training is 0.0001, and for testing is 0.0033. The *x* axis is the time in days and the *y* axis denotes the probability of overall survival.

With the approach mentioned above, the seven-feature prediction model and a binary classifier have been applied to distinguish between the low- and high-risk groups for both training set (*n*=65) and testing set (*n*=57). Kaplan-Meier survival curves have been estimated and plotted in Figure 5. The log rank test shows significant difference between two groups. The *p* value on testing set is slightly larger than the training set. The good performance demonstrates the accurate survival prediction power using this set of discovered image markers.

Using a multivariate Cox proportional hazards model, we have assessed the image markers related risk score in the context of other measured prognostic factors, including age, gender, cancer type, smoking history, and tumor stage. The results are presented in Table 5 and Table 6. The *p* value of Wald test of each covariate coefficient suggests that NSCLC subtype and tumor stage are significantly related to survival rate in the multivariate Cox regression without the image marker related risk score. However, when the image marker related risk score is introduced, it becomes the most significant variable in the model. To further quantify how much improvement is gained in survival analysis after the risk score is added, Akaike Information Criterion (AIC) and Bayesian Information Criterion (BIC) of these two models are computed. The experiments show that AIC=110.20, BIC=115.54 for the first model compared to AIC=99.81, BIC=106.04 for the model including the risk score. The clear difference demonstrates strong evidence in favor of the prognostic model with image marker related risk score. Hazard ratio is also measured and reported in Table 5 and Table 6. A hazard ratio greater than one, or equivalently, a value of coefficient greater than zero, indicates that as the value of this covariate increases, the event hazard increases and thus the length of survival decreases. Given the proposed comprehensive prediction model, for each NSCLC cancer patient with H&E stained diagnostic pathology image and clinical information, we can offer a personalized survival function and automatically group the individual into low- or high-risk group with an estimated risk score.

**Table 5:** **(TCGA NSCLC testing data** ***n*** **= 57) Multivariate Cox proportional hazards analysis of all clinical covariates without the image feature related risk score**

Variable	p-value	Hazard Ratio	SE (coef)
Age	0.0900	0.94	0.035
NSCLC subtype	0.0091	0.13	0.780
Smoking history	0.3914	0.80	0.479
Gender	0.7928	0.45	0.733
Tumor stage II	0.6800	1.46	0.924
Tumor stage III & IV	0.0076	0.05	1.138

**Table 6:** **(TCGA NSCLC testing data** ***n*** **= 57) Multivariate cox proportional analysis of all clinical covariates and image feature related risk score**

Variable	p-value	Hazard Ratio	SE (coef)
Risk score	0.0049	4.99	0.571
Age	0.1600	0.94	0.037
NSCLC subtype	0.0076	0.11	0.832
Smoking history	0.6530	0.72	0.741
Gender	0.4000	0.53	0.739
Tumor stage II	0.6000	1.64	0.930
Tumor stage III & IV	0.0090	0.04	1.160

Smoking history is a continuous variable representing years of smoking history. Gender is a binary variable (0 for male and 1 for female). Cancer type is also a binary variable (0 for squamous cell carcinoma and 1 for adenocarcinoma). Tumor stage is a three level categorical variable (stage I is treated as the reference group).

To measure the robustness of the feature selection, we have conducted the bootstrap analysis. We have resampled the whole dataset 5000 times with replacement, performed the boosting feature selection procedure on each sample and counted the frequency of selecting one specific feature variable. The top 10 most frequently selected image markers are: peri6, homo3, homo4, homo5, skew6, lbp5, lbp16, csac6, csac15 and tfcm18. Among the top 10 image features that are most highly associated with survival, 4 are pixel intensity features, 5 are image texture features and only 1 belongs to geometric feature. Moreover, 4 out of the 7 significant features previously selected in the training set are from the top 11 features in bootstrap analysis on the whole set, which shows good consistence of the proposed algorithm.

**Figure 6:**
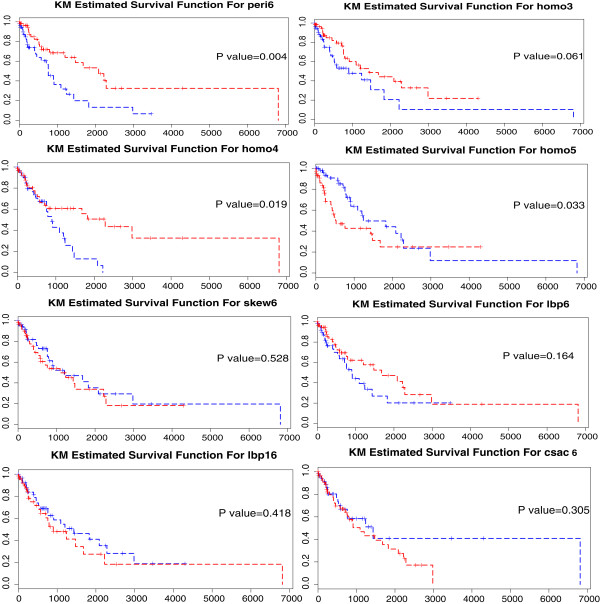
**Kaplan-Meier survival curves of the developed prognostic model for TCGA NSCLC dataset.** High value (great than or equal to median) groups are plotted as red lines, and low value (less than median) groups are plotted as blue lines. The *x* axis represents the time in days and *y* axis denotes the probability of overall survival. (R1C1) Survival curves of two groups with distinct values of feature peri6 with a log rank *p* value = 0.004. (R1C2) Survival curves of two groups with distinct values of feature homo3 with a log rank *p* value = 0.061. (R2C1) Survival curves of two groups with distinct values of feature homo4 with a log rank *p* value = 0.019. (R2C2) Survival curves of two groups with distinct values of feature homo5 with a log rank *p* value = 0.033. (R3C1) Survival curves of two groups with distinct values of feature skew6 with a log rank *p* value = 0.528. (R3C2) Survival curves of two groups with distinct values of feature lbp6 with a log rank *p* value = 0.164. (R4C1) Survival curves of two groups with distinct values of feature lbp16 with a log rank *p* value = 0.418. (R4C2) Survival curves of two groups with distinct values of feature csac6 with a log rank *p* value = 0.305.

Univariate survival analysis has been conducted to validate the feature variable selection procedure by showing that the selected features are closely related to lung cancer patients’ survival time. We choose the median of each image marker as the threshold to group the patients and plot the Kaplan-Meier curves for those two groups. Log rank test is conducted to test the difference of the two curves. It is shown that 4 out of the top 8 image markers selected from the bootstrap analysis can achieve significant log rank test outcome at *α*=0.10 level while the others is still acceptable even though they do not reach statistical significance for this naive approach (Figure 6). In addition, no single image marker can achieve the same prediction power as the combined risk score using the set of discovered image markers.

## Discussion and conclusions

In this paper, we have investigated novel image markers for both computer aided diagnosis and prognosis of non-small cell lung cancer. We propose an integrated framework that consists of cell detection, segmentation, feature extraction, classification, discovery of image markers, and survival analysis for NSCLC. A multi-scale distance map-based voting algorithm is first introduced to localize individual cells, and a repulsive deformable model is proposed to segment the cells using the previous detection results as initializations. A complete set of cellular features are extracted, and several advanced classification techniques are compared using the image markers calculated in previous steps. Finally, a Cox model fitted with component-wise likelihood based boosting is applied and several survival analysis approaches have been conducted to evaluate the discovered image features. Through extensive experiments, we have found a set of diagnostic image markers that are highly correlated to NSCLC subtype classification. In addition, we have also discovered a set of prognostic image markers (majorly representing image staining characteristics and inhomogeneity inside the nuclei of cancer cells) to predict NSCLC patients’ survival. We statistically prove that the developed comprehensive image marker related risk score is a strong predictor for patients’ survival than traditional clinical factors. Together with clinical information, it provides significant clinical values for patients’ prognosis.
